# Downregulation of miR-204 expression defines a highly aggressive subset of Group 3/Group 4 medulloblastomas

**DOI:** 10.1186/s40478-019-0697-3

**Published:** 2019-04-03

**Authors:** Harish Shrikrishna Bharambe, Raikamal Paul, Pooja Panwalkar, Rakesh Jalali, Epari Sridhar, Tejpal Gupta, Aliasgar Moiyadi, Prakash Shetty, Sadaf Kazi, Akash Deogharkar, Shalaka Masurkar, Kedar Yogi, Ratika Kunder, Nikhil Gadewal, Atul Goel, Naina Goel, Girish Chinnaswamy, Vijay Ramaswamy, Neelam Vishwanath Shirsat

**Affiliations:** 1 Shirsat Laboratory, Advanced Centre for Treatment, Research & Education in Cancer, Tata Memorial Centre, Kharghar, Navi Mumbai, 410210 India; 2 Department of Radiation Oncology, Tata Memorial Centre, Kharghar, Navi Mumbai, 410210 India; 3 Bioinformatics Centre, Advanced Centre for Treatment, Research & Education in Cancer, Tata Memorial Centre, Kharghar, Navi Mumbai, 410210 India; 4 Department of Radiation Oncology, Tata Memorial Hospital, Tata Memorial Centre, Parel, Mumbai, 400012 India; 5 Department of Pathology, Tata Memorial Hospital, Tata Memorial Centre, Parel, Mumbai, 400012 India; 6 Department of Surgical Oncology, Tata Memorial Hospital, Tata Memorial Centre, Parel, Mumbai, 400012 India; 7 Department of Medical Oncology, Tata Memorial Hospital, Tata Memorial Centre, Parel, Mumbai, 400012 India; 8 Department of Neurosurgery, Seth G. S. Medical College & K. E. M. Hospital, Parel, Mumbai, 400012 India; 9 Department of Pathology, Seth G. S. Medical College & K. E. M. Hospital, Parel, Mumbai, 400012 India; 10 Homi Bhabha National Institute, Training School Complex, Anushakti Nagar, Mumbai, 400085 India; 11 Division of Haematology/Oncology, Department of Paediatrics, Hospital for Sick Children and University of Toronto, 555 University Ave, Toronto, ON M5G 1X8 Canada

**Keywords:** Medulloblastoma, MiR-204, Risk stratification, Tumor-suppression, Autophagy

## Abstract

Genome-wide expression profiling studies have identified four core molecular subgroups of medulloblastoma: WNT, SHH, Group 3 and Group 4. Molecular markers are necessary for accurate risk stratification in the non-WNT subgroups due to the underlying heterogeneity in genetic alterations and overall survival. MiR-204 expression was evaluated in molecularly classified 260 medulloblastomas from an Indian cohort and in 763 medulloblastomas from the MAGIC cohort, SickKids, Canada. Low expression of miR-204 in the Group 3 / Group 4 tumors identify a highly aggressive subset of tumors having poor overall survival, in the two independent cohorts of medulloblastomas. Downregulation of miR-204 expression correlates with poor survival within the Group 4 as well indicating it as a valuable risk-stratification marker in the subgroup. Restoration of miR-204 expression in multiple medulloblastoma cell lines was found to inhibit their anchorage-independent growth, invasion potential and tumorigenicity. IGF2R was identified as a novel target of miR-204. MiR-204 expression resulted in downregulation of both M6PR and IGF2R that transport lysosomal proteases from the Golgi apparatus to the lysosomes. Consistent with this finding, miR-204 expression resulted in reduction in the levels of the lysosomal proteases in medulloblastoma cells. MiR-204 expression also resulted in inhibition of autophagy that is known to be dependent on the lysosomal degradation pathway and LC3B, a known miR-204 target. Treatment with HDAC inhibitors resulted in upregulation of miR-204 expression in medulloblastoma cells, suggesting therapeutic role for these inhibitors in the treatment of medulloblastomas. In summary, miR-204 is not only a valuable risk stratification marker in the combined cohort of Group 3 / Group 4 medulloblastomas as well as in the Group 4 itself, that has paucity of good prognostication markers, but also has therapeutic potential as indicated by its tumor suppressive effect on medulloblastoma cells.

**Electronic supplementary material:**

The online version of this article (10.1186/s40478-019-0697-3) contains supplementary material, which is available to authorized users.

## Introduction

Brain tumors are the second most common cancers in children and the leading cause of cancer-related mortality in this age group [40]. Medulloblastoma, a highly malignant tumor of the posterior fossa region of the brain, is the single most common pediatric malignant brain tumor. Genome-wide expression profiling studies have identified four core molecular subgroups of medulloblastomas: WNT, SHH, Group 3 and Group 4 that are not only distinct in their underlying genetic alterations but also differ in clinical characteristics like age, gender related incidence, incidence of metastasis and overall survival rates [36]. WNT subgroup medulloblastomas that are characterized by the activation of the canonical WNT signaling pathway have excellent (> 95%) long term survival [44]. SHH subgroup medulloblastomas having activated Sonic Hedgehog signaling expression profile, have intermediate survival rates with those harboring mutation in the *TP53* tumor suppressor gene or amplification of *MYCN* oncogene having poor survival [44]. The two non-WNT, non-SHH subgroups have some overlap in their expression profiles with a number of transcription factors involved in neural development being overexpressed in both the subgroups [37]. The two subgroups are distinguished based on the preferential expression of proliferation related genes, retina-specific genes in the Group 3 tumors and neuronal differentiation related genes in the Group 4 tumors [37]. Group 3 tumors have the worst survival rates among all the four subgroups while Group 4 tumors have intermediate survival rate. NRL and CRX, the two retina-specific transcription factors have been found to be master regulators of photoreceptor signaling program in the Group 3 medulloblastomas [9]. MYC amplifications are restricted to Group 3 [38]. Structural variants leading to aberrant induction of *GFI1/GFI1B* oncogenes and *MYCN* amplifications are found in both Group 3 and Group 4. Pathway analysis of recurrent genetic alterations have found overrepresentation of genes involved in the TGFβ and Notch signaling pathway in Group 3 and chromatin modifiers in Group 4 [34].

Surgery followed by radiation therapy and chemotherapy is the standard multimodal treatment for medulloblastoma [1]. Long term sequelae of the intense treatment include neurocognitive impairment, endocrine dysfunction, psychiatric, developmental deficits and in some cases secondary malignancies [17]. Accurate risk stratification of medulloblastomas is therefore necessary to spare the children having low risk of recurrence from excessive treatment to the developing brain. On the other hand, survival of high risk medulloblastoma cases can be improved by more aggressive treatment. Considerable heterogeneity exists in each of the three non-WNT subgroups for which molecular markers are necessary so that accurate risk stratification can be done for effective treatment with least side effects [44].

MicroRNAs are small non-coding molecules that have been shown to regulate a wide array of cell functions, ranging from cell proliferation, differentiation, cell death and stress resistance. Since the first report of miR-15/miR-16 deletion in B cell chronic lymphocytic leukemia, large number of studies have reported microRNA dysregulation in cancer including medulloblastoma [5, 56]. We have earlier reported differential expression profiles of microRNAs in the molecular subgroups of medulloblastomas [13]. Further, we have developed an assay based on the microRNA profile that has 97% accuracy for molecular classification of medulloblastomas and is particularly useful for formalin-fixed, paraffin-embedded (FFPE) tumor tissues [19]. In the present study, miR-204 expression was analyzed in 260 medulloblastomas from an Indian cohort and in 763 medulloblastomas from the MAGIC (Medulloblastoma Advanced Genomics International Consortium) cohort [2]. A subset of Group 3 / Group 4 medulloblastomas having low expression of miR-204 was found to have significantly poor survival. The role of miR-204 expression in medulloblastoma biology was investigated by restoring miR-204 expression in established medulloblastoma cell lines and studying its effect on growth and malignant behavior of medulloblastoma cells.

## Materials and methods

### Human tissue samples

Medulloblastoma tumor tissues either as fresh frozen or FFPE tissues were obtained after acquiring informed consent from the patients. The study was approved by the Institutional Ethics Committee of the Tata Memorial Centre. The tumor tissues were snap-frozen in liquid nitrogen immediately after surgical resection and stored at − 80 °C. The histopathological diagnosis and grading of the tumor tissues was done as per the World Health Organization 2007 classification of tumors of the Central Nervous System [25] and only the tumors diagnosed as medulloblastomas were included in the study. Normal human brain tissues were obtained from the Human Brain Tissue Repository at the National Institute of Mental Health and Neurosciences, Bengaluru, India.

### Analysis of miR-204 expression

Molecular classification of 260 medulloblastomas from the Indian cohort was carried out using real time RT-PCR (Reverse Transcription-Polymerase Chain Reaction) assay as described before [19]. MiR-204 expression was determined by the Taqman assay. *RNU48* was used as a house-keeping small RNA control. Relative Quantity (RQ) was estimated as RQ = 2^- (Ct^_test_^– Ct^_control_^)^ X 100. In the MAGIC validation cohort, miR-204 expression was analyzed across 763 primary medulloblastoma samples, profiled on the Affymetrix Gene 1.1 ST array as described previously, normalized using the RMA (Robust Multi-array Average) method and, subgrouped / subtyped using similarity network fusion (GSE85217) [2]. Differences across subgroups and subtypes were evaluated using ANOVA (Analysis of variance) in the R statistical environment (v3.4.2). Survival was measured from the time of initial diagnosis to the date of death or last follow up. Survival distribution was estimated according to the Kaplan–Meier method using optimal cut-off selection and log-rank statistics using the survival package (v2.40–1) in the R statistical environment (v3.4.2). *P* values < 0.01 were considered to be statistically significant.

### Cell culture

Human medulloblastoma cell line D283 was obtained from ATCC (American Type Culture Collection), Manassas, VA, USA. Authenticity of the cell lines was confirmed by the Short Tandem Repeat (STR) marker profiling before initiating the experiments. Medulloblastoma cell lines D425, D341 are kind gifts from Dr. Darell Bigner, Duke University Medical Centre, Durham, NC, USA. HD-MB03 cell line is a kind gift from Dr. Till Milde, German Cancer Research Centre, Germany. All the cell lines were checked for the presence of mycoplasma contamination by PCR based assay [53]. The cells were grown in Dulbecco’s Modified Eagle Medium: Nutrient Mixture F-12 (DMEM/ F-12) supplemented with 10% Fetal Bovine Serum (FBS) in a humidified atmosphere of 5% CO_2_.

### Restoration of miR-204 expression in medulloblastoma cells

Genomic region encoding miR-204 was amplified from normal human lymphocyte DNA by PCR and cloned in pTRIPZ lentiviral vector downstream of doxycyline-inducible minimal Cytomegalo virus (CMV) promoter (Additional file 1: Table S1). The medulloblastoma cell lines were transduced with the pTRIPZ-miR-204 lentiviral particles and stable polyclonal populations were selected in the presence of puromycin. The cells transduced with lentiviral particles of empty pTRIPZ vector (Dharmacon, Lafayette, CO, USA) were used as vector control.

### Effect of miR-204 expression on proliferation and anchorage-independent growth

Growth of miR-204 expressing cells and control cells was studied by the MTT reduction assay as described before [32, 59]. 2000 cells of the medulloblastoma cell lines were seeded per well of a 96-well micro-titer plate. Cell growth was followed over a period of 10-12 days with replenishment of medium every 3^rd^ day. For studying anchorage-independent growth by soft agar colony formation assay, 2000 cells were seeded in DMEM/F12 medium supplemented with 10% FBS containing 0.3% agarose over a basal layer of 1% agarose in DMEM-F12/10% FBS. The cells were incubated for about 1-2 weeks and the colonies formed were counted.

### Invasion assay

75,000 cells of D283 / HD-MB03 cell line were seeded in 200 μl of serum-free DMEM / F12 medium in the upper chamber of 8-μm pore size transwell inserts (BD Biosciences, San Hose, CA, USA) coated with Matrigel™, placed in a 24 well micro-titre plate. 750 μl of the medium supplemented with 10% FBS was added to the lower chamber. The cells were allowed to migrate for 56 h to 72 h depending upon the cell line and then labeled with Calcein-AM (Life technologies, Carlsbad, CA, USA), a fluorescent dye, 30 min prior to terminating the invasion. Non-invaded cells from the upper chamber were removed by wiping the upper portion of the insert with a cotton bud. The inserts were photographed using a Zeiss Axiovert 200 M fluorescence microscope. Fluorescence intensity of the Calcein-AM labeled cells on the lower side of the insert was measured using a Mithras LB940 multimode reader (Berthhold Technologies, Bad Wildbad, Germany) using excitation wavelength of 485 nm and emission wavelength of 535 nm.

### Tumorigenicity assay

The experimental protocols were approved by the Institutional Animal ethics committee. Medulloblastoma cells were transduced with lentiviral particles of pCS-CG vector (a gift from Inder Verma, Addgene plasmid #12154 [30]) expressing firefly luciferase cDNA FL2 (from pCAG-luciferase vector, a gift from Snorri Thorgeirsson, Addgene plasmid #55764 [21]) under the CMV promoter. 2 X 10^5^ doxycycline-induced cells were injected into the cerebellum of NOD/SCID mice (NOD.CB17*-Prkdc*^*scid*^/NCrCrl, Charles River, USA) through 0.5 mm burr hole in the midline, 2 mm posterior to lambda at 2 mm depth, using small animal stereotaxic frame under anesthesia [59]. Tumor growth was monitored by in vivo bioluminescence imaging using the IVIS Spectrum imaging system (Caliper Lifesciences, Perkin Elmer, MA, USA). Tumor bearing mice were maintained until they succumbed to the tumor or were about to succumb to the tumor as judged by over 40% loss of weight or other clinical symptoms. Upon sacrifice, whole brain was fixed in the neutral buffered formalin and embedded in a paraffin block. Hematoxylin & Eosin stained sections of the paraffin blocks were used for determination of the invasive capacity of the tumor cells without revealing identity of the specimen to the analyst.

### Transcriptome sequencing

Libraries were prepared using the Truseq RNA sample prep kit V2 as per the manufacturer’s protocol (Illumina, San Diego, USA) from the total RNA extracted from the medulloblastoma cells and subjected to 100 nucleotides deep sequencing using the Illumina HiSeq 2500 sequencing system to get a minimum of 10 million reads per library. The reads were aligned to the reference human genome hg19 using the TopHat version 2.0.13 (http://ccb.jhu.edu/software/tophat) with default parameters. Raw counts for the reads aligned to the gene intervals were produced by the python package HTSeq version 0.6.1 (www-huber.embl.de/users/anders/HTSeq) using the default union-counting mode. The data was normalized by variance stabilizing transformation using the DESeq software that takes into account RNA-seq data size of each sample (http://bioconductor.org/packages/release/bioc/html/DESeq.html). Gene Set enrichment analysis of the genes differentially expressed upon miR-204 expression was done using the GSEA (Gene Set Enrichment Analysis) software (software.broadinstitute.org/gsea/index.jsp). Downregulation of expression of known miR-204 target genes upon miR-204 expression in medulloblastoma cell lines was validated by SYBR green real time RT-PCR assay using gene-specific primers (Additional file 1: Table S1).

### Western blotting

Total protein extracted from the medulloblastoma cells was separated by SDS-PAGE electrophoresis, blotted onto a PVDF membrane (Merck Millipore, Berlington, MA, USA) and probed with the primary antibody as per the manufacturer’s protocol. The images were captured using the ChemiDoc gel imaging system (Biorad Hercules, CA, USA) or by autoradiography. The captured images were quantified using the Image Lab software (Bio-Rad, Hercules, CA, USA) or ImageJ software (imajeJ.nih.gov.in). The antibodies used for the Western blotting experiments are listed below.

I. Anti-LC3B (#2775), anti-p62/SQSTM1 (#8025), anti-Cathepsin D (#2284), anti-Cathepsin B (#31718) and, anti-IGF2R (#14364) antibodies from the Cell signaling technology, Boston, MA, USA.
II. Anti-GAPDH antibody (SC 47724) from Santa Cruz Biotechnology, Dallas, TX, USA.
III. Anti-Histone H3 (acetyl K9) antibody (ab10812) from Abcam, Cambridge, UK.

### Luciferase reporter assay

Firefly luciferase cDNA was cloned in the pcDNA 3.0 vector (Invitrogen, Carlsbad, CA, USA) downstream of the CMV promoter to generate ‘pLuc’ reporter vector. 3′-UTR regions of the miR-204 target genes were amplified from the genomic DNA of normal human lymphocytes and cloned downstream of the firefly luciferase cDNA in the ‘pLuc’ vector. Putative miR-204 binding sites in the 3′-UTRs were mutated by site-directed mutagenesis using primers having 4 nucleotides corresponding to the binding site altered [59]. Luciferase activity was assessed from the HEK293FT cells transfected with the luciferase reporter plasmid, miR-204 expressing plasmid/vector control pcDNA4 (Invitrogen, Carlsbad, CA, USA), and a plasmid vector expressing EGFP fluorescent protein. Luciferase activity was assessed from the total protein extracted from the transfected HEK293FT cells and was normalized against the EGFP fluorescence measured using the BioTek Cytation Hybrid Multimode Reader, Winooski, VT, USA.

### *TRPM3/MIR-204* promoter methylation analysis and upregulation of miR-204 expression upon treatment with histone deacetylase inhibitors

Genomic DNA was isolated from the medulloblastoma cell lines using QIAamp DNA mini kit (Qiagen, GmbH, Hilden, Germany) as per the manufacturer’s instructions. Bisulfite conversion of 500 ng of the genomic DNA was performed using EZ DNA Methylation-Gold Kit from Zymo Research, Irvine, CA, USA, as per the manufacturer’s instructions. The 203 bp region covering - 200 to + 3 with respect to the known transcription start site of the *TRPM3* gene was PCR amplified using the primers designed to amplify bisulfate converted DNA and sequenced (Additional file 1: Table S1). Medulloblastoma cells were treated with HDAC inhibitors Sodium valproate (6 mM) and Trichostatin A (400 nM) for a period of 16 h. Histone acetylation status was evaluated by separating total protein extracts from the treated cells by SDS-PAGE and probing the Western blot using anti-H3K9 acetylation antibody .

All experiments were performed at least three times and the Student’s t-test was used for evaluating statistical significance of the difference in the test as compared to the control. Error bars indicate standard error of the mean/median.

## Results

### MiR-204 downregulation identifies a highly aggressive subset of Group 3 / Group 4 medulloblastomas having poor survival

**Fig. 1:**
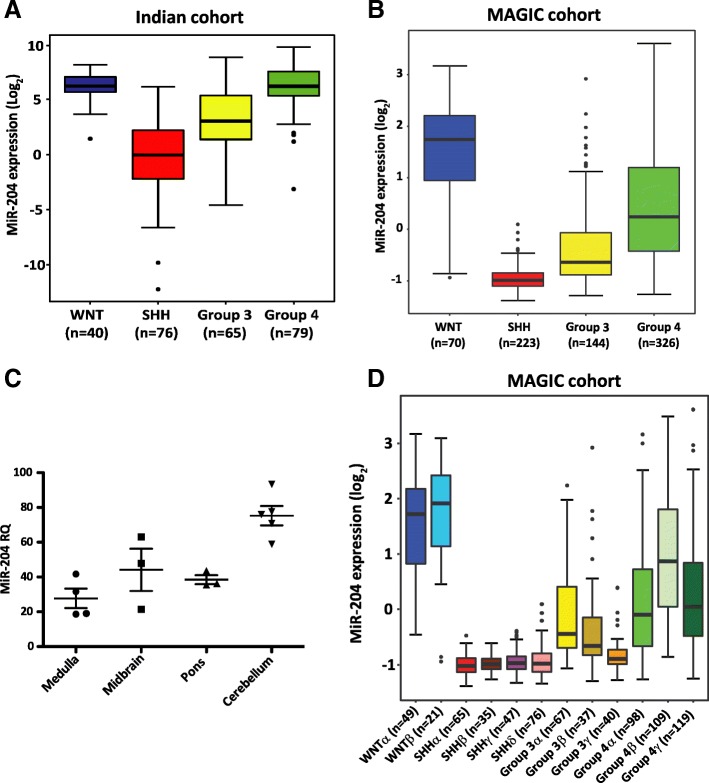
MiR-204 expression across molecular subgroups/subtypes of medulloblastoma and in normal brain tissues. MiR-204 expression levels in the four subgroups of medulloblastomas from the Indian cohort (**a**, *n* = 260) and the MAGIC cohort (**b**, *n* = 763). **c**. MiR-204 expression in the normal brain tissues from the posterior fossa region, evaluated by the Taqman assay. **d**. MiR-204 expression in the 12 subtypes of medulloblastomas from the MAGIC cohort (*n* = 763)

MiR-204 expression was studied in an Indian cohort of 260 medulloblastomas and a non-overlapping MAGIC cohort of 763 medulloblastomas [2]. MiR-204 was found to be differentially expressed in the four molecular subgroups, with all WNT and 75-84% of Group 4 tumors having high miR-204 (RQ > 20) while expression is low in almost all SHH subgroup tumors and in 54-76% of the Group 3 medulloblastomas (Fig. 1a, b). MiR-204 expression in the normal posterior fossa brain regions: cerebellum, mid-brain, pons and medulla, that are believed to be the sites of origin for the 4 molecular subgroups of medulloblastoma [11], was found to range from RQ = 21 to 93 (Fig. 1c). Thus, miR-204 expression is downregulated in the SHH subgroup and in a subset of the Group 3/Group 4 medulloblastomas. Integrated analysis of the genome wide DNA methylation data, expression data and copy number alterations data, has identified 12 subtypes corresponding to the four core subgroups of medulloblastomas [2]. MiR-204 expression levels were found to be high in both the WNT subtypes, low in all four SHH subtypes, low in all three Group 3 subtypes and low to moderate in two out of 3 subtypes of Group 4 (Fig. 1d). Group 3γ having the worst outcome [2] has the least miR-204 expression among the Group 3 / Group 4 subtypes. Highly integrative analysis that integrated somatic mutation data analysis in addition to the genome wide methylation, transcriptome and copy number variation data has reported 8 subtypes within the Group 3/Group 4 medulloblastomas [34]. Analysis of MiR-204 expression in these 8 subtypes showed that the expression levels vary across the 8 subtypes with the least expression in the 3 subtypes (ii, iii and iv) that contain only Group 3 tumors (Additional file 2: Figure S1). The subtype ii enriched for MYC amplification has the least miR-204 expression levels. MYC amplification is a known marker for poor prognosis in the Group 3 medulloblastomas [44].

**Fig. 2:**
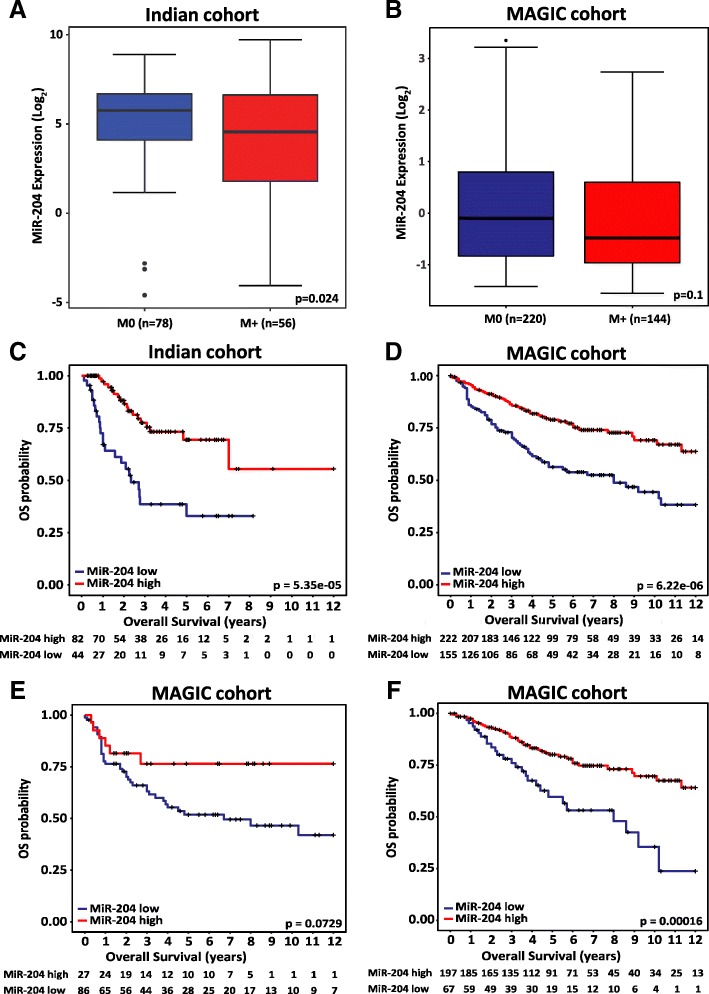
Correlation of miR-204 expression with metastasis at diagnosis and overall survival. MiR-204 expression in Group 3 / Group 4 medulloblastomas having presence (M+) or absence (M0) of metastasis at diagnosis in the Indian cohort (**a**) and in the MAGIC cohort (**b**). Kaplan Meier survival analysis comparing overall survival of ‘MiR-204 low’ subset with that of ‘MiR-204 high’ subset of the Group 3 / Group 4 medulloblastomas from the Indian cohort (**c**) and from the MAGIC cohort (**d**). Kaplan Meier survival analysis comparing overall survival of ‘MiR-204 low’ with that of ‘MiR-204 high’ subset of Group 3 (**e**) and Group 4 (**f**) medulloblastomas from the MAGIC cohort

Group 3 / Group 4 medulloblastomas in the Indian cohort having metastasis at diagnosis were found to have significantly (*p* = 0.024) lower miR-204 expression (Fig. 2a). In the MAGIC cohort as well, miR-204 expression levels are lower in the Group 3 / Group 4 tumors having metastasis at diagnosis, although the difference is not statistically significant (Fig. 2b). Low miR-204 expression in the Group 3 / Group 4 medulloblastomas was found to correlate with poor overall survival in the Indian cohort (*n* = 126) as well as in the larger MAGIC cohort (*n* = 377) (Fig. 2c, d). Five year overall survival of the ‘miR-204 low’ subset in the Indian cohort is 33% (95% CI, 16.8-50.2%) as compared to 69.4% (95% CI, 54.1-80.4%) of the ‘miR-204 high’ subset. In the MAGIC cohort as well, five year survival of the ‘miR-204 low’ subset is lower at 56.3% (95% CI 48.1% - 65. 9%) as compared to 78.9% (95% CI 73.1-85.2%) of the ‘miR-204 high’ subset. Thus, low miR-204 levels identify a subset of Group 3/Group 4 tumors having poor overall survival both in the Indian cohort and in the larger MAGIC cohort. In the MAGIC cohort within the Group 3 tumors, low expression levels of miR-204 have a trend towards worse survival, although it does not reach statistical significance due to lower fraction of ‘miR-204 high’ tumors (Fig. 2e). In the Indian cohort, low expression levels of miR-204 correlate with poor survival within the Group 3 as well (Additional file 3: Figure S2). Due to small proportion of ‘miR-204 low subset’ in the Group 4 tumors of the Indian cohort, survival analysis for the Group 4 was done only for the MAGIC cohort. The five year survival of the Group 4 ‘miR-204 high’ subset is 80% (95% CI 74-86.6%) while that of the ‘miR-204 low’ subset is 59.7% (95% CI 47.2-75.4%) in the MAGIC cohort (Fig. 2f). Low miR-204 expression levels thus, identify a subset having poor overall survival within the Group 4 itself as well.

### Restoration of miR-204 expression inhibits anchorage-independent growth, and tumorigenicity of medulloblastoma cells

**Fig. 3:**
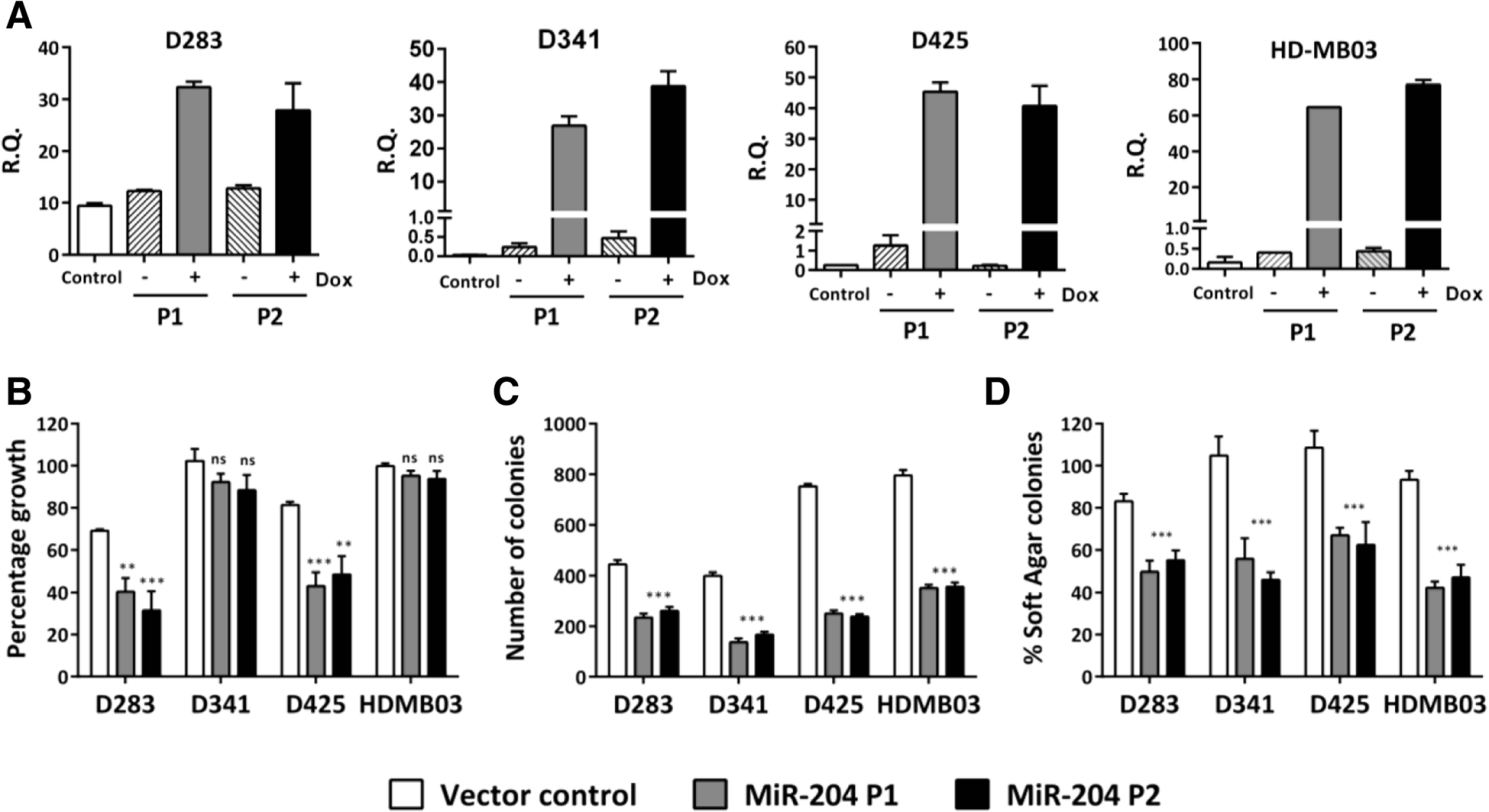
MiR-204 expression levels in the medulloblastoma cell lines before and after exogenous expression and effect of restoration of miR-204 expression on proliferation and anchorage-independent growth. **a**. MiR-204 expression in the parental cells (control) and the established polyclonal populations P1, P2 of the indicated medulloblastoma cell line with (+) and without (−) doxycycline induction. **b**. Effect of miR-204 expression on growth of medulloblastoma cells studied by the MTT assay. Y-axis denotes growth of the vector control and polyclonal populations P1, P2 of the indicated medulloblastoma cell line after doxycycline treatment as a percentage of the growth of the corresponding un-induced population of the cells. **c** & **d**. Effect of miR-204 expression on the anchorage-independent growth of medulloblastoma cell lines studied by the soft agar colony formation assay. Y axis denotes the number of soft agar colonies formed by the P1, P2 polyclonal populations and the Vector control population of the indicated cell line upon doxycycline induction of miR-204 expression (**c**) and in (**d**), as a percentage of those formed by the un-induced cells. **, *** indicate *p* ≤ 0.001 and *p* ≤ .0001 respectively

MiR-204 expression levels in the established medulloblastoma cell lines D341, D425 and HD-MB03 were found to be in the range of RQ = 0.02 to 0.14 (Fig. 3a). D341, D425 and the recently established HD-MB03 cell line belong to the Group 3 [16, 29]. MiR-204 expression in the D283 cell line which has characteristics intermediate between Group 3 and Group 4 is at RQ = 9.4 ± 1.0 (Fig. 3a) [16]. The cell lines were transduced with the pTRIPZ lentiviral vector expressing miR-204 in a doxycycline inducible manner. Stable polyclonal populations of the four medulloblastoma cell lines express miR-204 at levels (RQ = 25 to 70) comparable to that in the normal brain tissues after induction with doxycycline (Fig. 3a). Effect of miR-204 expression on the proliferation and anchorage-independent growth of these cell lines was studied by the MTT assay and soft agar colony formation assay respectively. While miR-204 expression inhibited proliferation of D283 and D425 cells by 25 to 40%, it did not affect proliferation of D341 and HD-MB03 medulloblastoma cells (Fig. 3b). MiR-204 expression resulted in significant inhibition of soft agar colony formation capacity (35 to 55%, *p* < 0.001) of all the four medulloblastoma cell lines studied (Fig. 3c, d).

**Fig. 4:**
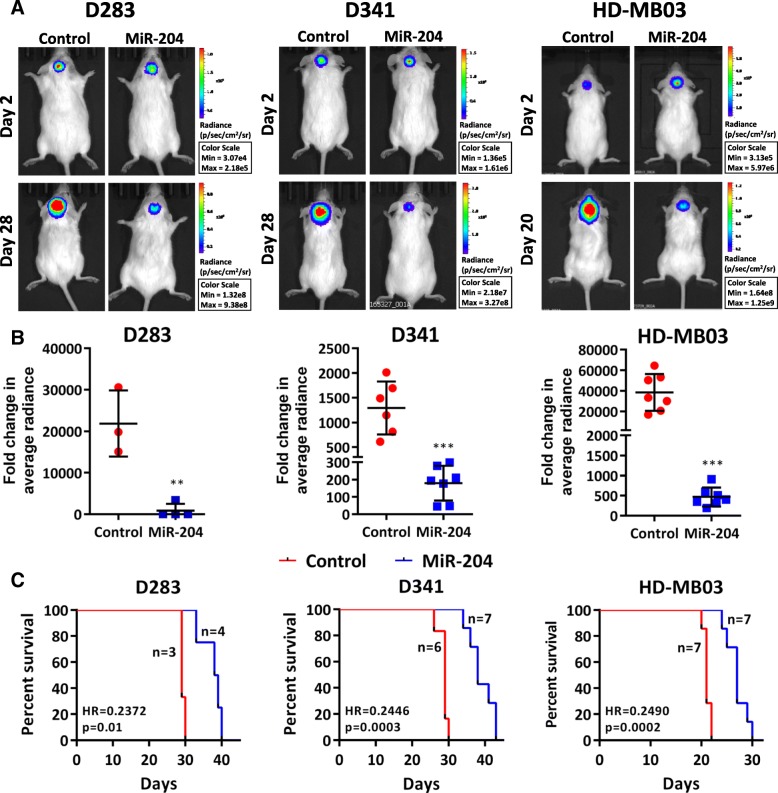
Effect of miR-204 expression on tumorigenicity of the medulloblastoma cells studied by monitoring orthotopic xenograft development. NOD/SCID mice were orthotopically injected with doxycycline treated vector control and P1 population expressing miR-204 of D283, D341 and HD-MB03 cells. **a**. Bioluminescence images of the injected mice on the indicated day post-injection of the indicated cell line. **b**. Y-axis denotes the fold increase in the average radiance of the tumor luminescence on day 28 or day 20 as compared to day 2 post-injection of the indicated cell population. **c**. Kaplan Meier Survival analysis of the injected mice. Significance in the difference in the survival upon miR-204 expression was determined by the Log Rank test. HR: Hazard Ratio; **, *** indicate *p* ≤ 0.001 and *p* ≤ .0001 respectively

In order to study the effect of miR-204 expression on tumorigenic potential, D283, D341, HD-MB03 cells as well as their miR-204 expressing polyclonal population cells were engineered to express firefly luciferase and, were injected stereotactically in cerebellum of NOD/SCID mice after doxycycline induction. MiR-204 expression was found to significantly (*p* < 0.002 to 0.0001) decrease tumorigenicity of all the 3 medulloblastoma cell lines as judged by the in vivo imaging of the orthotopic tumors (Fig. 4a). The tumor volume decreased by 8.8-fold to 25-fold upon miR-204 expression (Fig. 4b). Further, survival of the tumor bearing mice increased by 26 to 34% upon miR-204 expression (Fig. 4c) in all the three medulloblastoma cell lines studied.

### MiR-204 expression inhibits invasion potential of medulloblastoma cells in vitro and in vivo

**Fig. 5:**
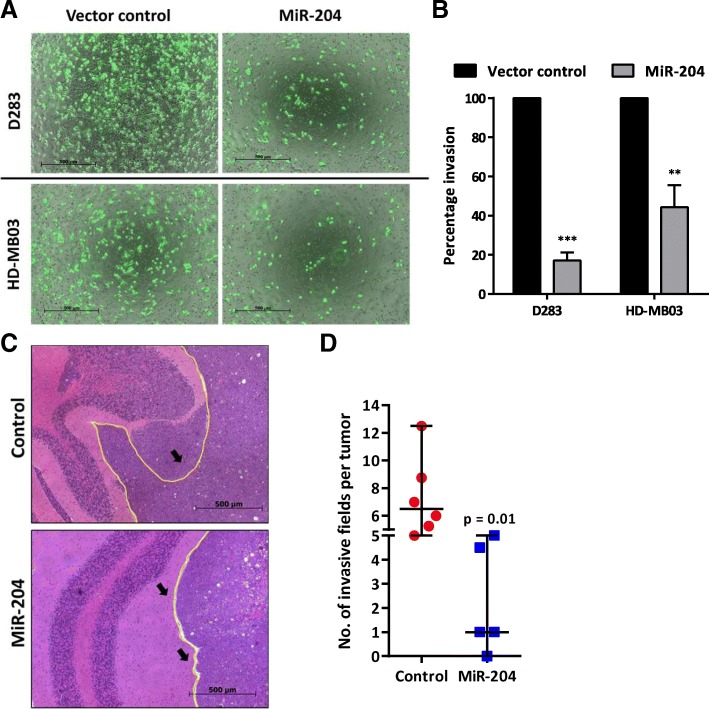
Effect of miR-204 expression on in vitro and in vivo invasion potential of medulloblastoma cells. **a**. Representative Images of the Calcein-labeled cells on the lower side of the transwell insert membrane, post-invasion of the doxycycline treated miR-204 expressing or vector control polyclonal population of D283 and HD-MB03 cells. **b**. Y- axis indicates the percentage of the invaded cells of the miR-204 expressing polyclonal population as compared to the Vector Control population of the cells, evaluated by measuring fluorescence intensity of the invaded cells. **c**. Photographs of hematoxylin-eosin stained paraffin sections (sagittal section) of the orthotopic xenografts of doxycycline treated vector control cells and miR-204 expressing polyclonal population of D341 cells. Arrows show the vector control cells invading cerebellar folia boundary as compared to the cohesive margin of the miR-204 expressing cells. Yellow line indicates cerebellar folia margin. **d**. The scatter dot plot shows significant difference in the number of invasive fields per tumor in the miR-204 expressing tumors as compared to those expressing vector control D341 cells. **, *** indicate *p* ≤ 0.001 and *p* ≤ .0001 respectively

Effect of miR-204 expression on the invasion potential of medulloblastoma cells was studied by evaluating invasion of the cells through Matrigel™ coated membranes in transwell inserts. Figure 5a shows images of the Calcein-AM labeled medulloblastoma cells that have invaded the matrigel coated membrane after 56 h to 72 h. Evaluation of the fluorescence intensity of the invaded cells showed 60–80% reduction in the invasion potential of D283 and HD-MB03 cells upon miR-204 expression (Fig. 5b). Furthermore, invasive capacity of the medulloblastoma cells as judged by their in vivo invasion across the cerebellar folia boundary was found to be reduced upon miR-204 expression (Fig. 5c, d). Thus, miR-204 expression reduced invasion potential of the medulloblastoma cells both in vitro and in vivo.

### MiR-204 expression downregulates *M6PR*, *IGF2R* genes involved in the lysosomal pathway and inhibits autophagy of medulloblastoma cells

**Fig. 6:**
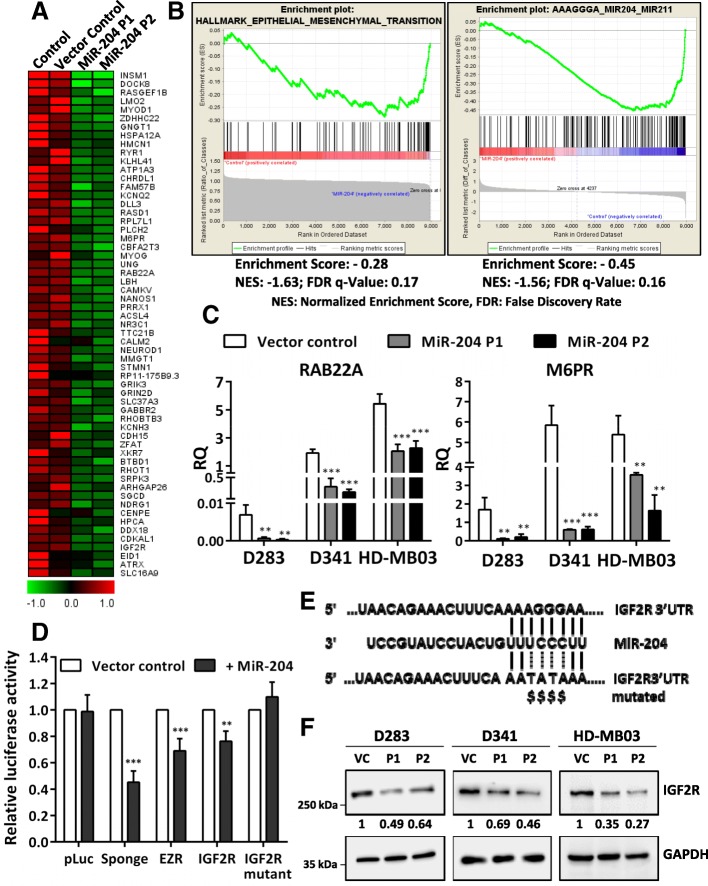
Downregulation of miR-204 target genes upon its expression in medulloblastoma cells. **a**. Heat map shows top 60 rank ordered genes (GSEA) whose expression is downregulated upon miR-204 expression in the two polyclonal populations, as compared to the doxycycline treated parental HD-MB03 cells and the vector control cells, identified by the RNA-seq analysis. Known validated miR-204 targets M6PR and RAB22A are indicated by arrows. **b**. GSEA analysis of the genes downregulated upon miR-204 expression in HD-MB03 cells showed most significant enrichment of the Epithelial Mesenchymal transition genes and the ‘miR-204 target gene set’ (software.broadinstitute.org/gsea/msigdb/collections.jsp). **c**. Real time RT-PCR analysis showing downregulation of the known miR-204 targets RAB22A, M6PR upon miR-204 expression in the medulloblastoma cell lines. **d**. Y axis shows luciferase activity of the indicated p-Luc construct upon co-transfection with miR-204 expressing plasmid relative to that obtained upon cotransfection with the Vector control. 3′-UTR of the *EZR* gene and a sponge construct containing six miR-204 binding sites were used as positive controls. **e** Nucleotide sequence of the miR-204 target site in the 3’UTR of the *IGF2R* gene and the mutations introduced indicated by ‘$’. **f**. Western blot analysis showing downregulation of IGF2R protein levels upon miR-204 expression in the polyclonal populations P1, P2 of the indicated medulloblastoma cell line as compared to the doxycycline treated vector control (VC) cells. The numbers below the western blot indicate fold change in the IGF2R protein levels upon miR-204 expression level using GAPDH levels as a house-keeping control for normalization. **, *** indicate *p* ≤ 0.001 and *p* ≤ .0001 respectively

In order to delineate the molecular mechanism underlying the tumor suppressive role of miR-204 in medulloblastoma cells, genes significantly differentially expressed upon miR-204 expression were identified by the RNA-seq analysis. Figure 6a shows a heat map of the top 60 genes downregulated upon miR-204 expression in HD-MB03 cells. GSEA analysis identified most significant enrichment of Epithelial Mesenchymal Transition genes in the genes downregulated upon miR-204 expression (Fig. 6b). GSEA analysis using the microRNA target motif database identified most significant enrichment of miR-204 targets in the genes downregulated upon miR-204 expression (Fig. 6b). Known validated targets of miR-204 like *RAB22A, M6PR* are at the top of the list whose downregulation upon miR-204 expression was further confirmed by real time RT-PCR in the three medulloblastoma cell lines (Fig. 6c). IGF2R was one of the putative miR-204 targets identified by the GSEA analysis. 3′-UTR regions of *IGF2R* and the known target *EZR* were cloned downstream of the luciferase cDNA in the pcDNA3.0 vector. Luciferase reporter assay showed inhibition of luciferase activity upon co-transfection of these 3′-UTR constructs with the vector expressing miR-204 in HEK293FT cells suggesting *IGF2R* as a direct target of miR-204 (Fig. 6d). MiR-204 mediated inhibition of the luciferase activity was lost upon site-directed mutagenesis of the miR-204 binding site in the 3′-UTR of *IGF2R,* validating it as a direct target of miR-204 (Fig. 6d, e). Furthermore, downregulation of IGF2R protein levels upon miR-204 expression was confirmed by the western blotting in all the three medulloblastoma cell lines (Fig. 6f).

**Fig. 7:**
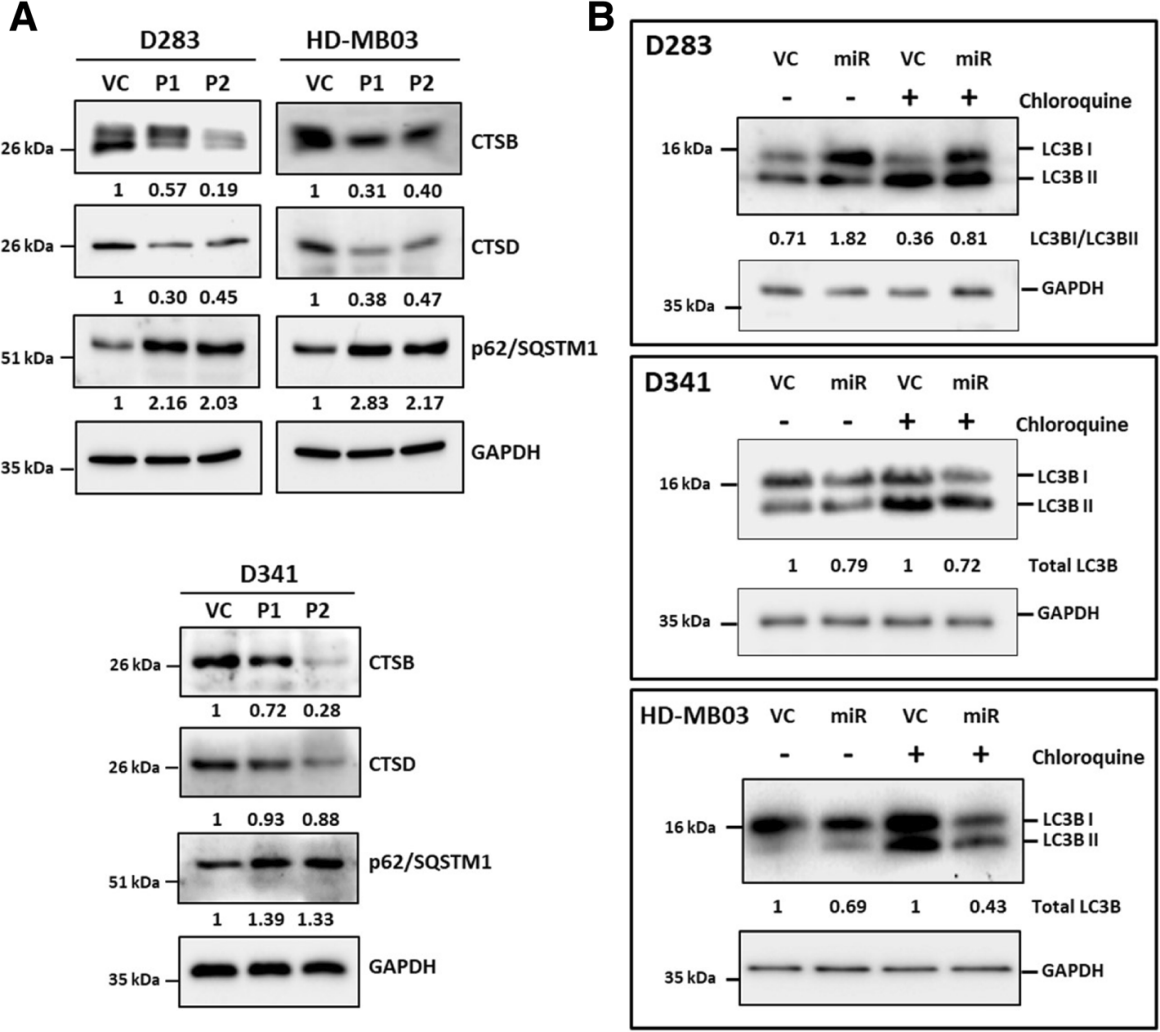
Effect of miR-204 expression on lysosomal enzymes and autophagy in medulloblastoma cells. **a**. Western blot analysis showing reduction in the levels of Cathepsin B (CTSB), Cathepsin D (CTSD) and increase in the levels of p62/SQSTM1 in the P1, P2 polyclonal populations of D283, D341 and HD-MB03 cells upon miR-204 expression. **b**. LC3BI, LC3BII levels in the miR-204 expressing polyclonal populations of D283, D341 and HD-MB03 cells as compared to the doxycycline treated vector control (VC) cells before and after treatment with Chloroquine for 1 h. The numbers below the western blots indicate the fold change in the indicated protein level or LC3BI / LC3B II ratio upon miR-204 expression using GAPDH as a house-keeping control

Both cation-dependent and cation-independent mannose-6-phosphate receptors i.e. M6PR and IGF2R respectively are known to be involved in trafficking of lysosomal proteases from the Golgi apparatus to lysosomes [26]. Therefore, effect of miR-204 expression on the levels of lysosomal enzymes Cathepsin B and Cathepsin D in medulloblastoma cells was studied by western blotting. MiR-204 expression resulted in considerable downregulation of these lysosomal enzymes in all three medulloblastoma cell lines D283, D341 and HD-MB03 (Fig. 7a). Lysosomal degradation pathway plays a major role in autophagy [12]. Besides, LC3B is a known target of miR-204 that plays a crucial role in autophagy [28]. Effect of miR-204 expression on autophagy was studied by evaluating LC3B flux. Upon autophagy induction, LC3BI isoform gets converted to LC3BII as a result of conjugation with phosphatidylethanolamine [12]. LC3BII levels however, decrease upon fusion of autophagosome to lysosome due to degradation by lysosomal enzymes. LC3B turnover is therefore studied in the presence and absence of an inhibitor of lysosomal degradation like chloroquine to evaluate LC3B flux [18]. MiR-204 expression resulted in high LC3BI / LC3BII ratio in D283 cells both before and after chloroquine treatment indicating low LC3B flux and thereby autophagy inhibition (Fig. 7b). In D341 and HD-MB03 medulloblastoma cells, total levels of LC3B decrease upon miR-204 expression both before and after treatment with chloroquine, indicating lower LC3B turnover and thereby autophagy inhibition (Fig. 7b). Autophagy inhibition is known to be accompanied by increase in the levels of p62/SQSTM1 adapter protein [12]. Expression levels of p62/SQSTM1 increased upon miR-204 expression in the three medulloblastoma cell lines further confirming autophagy inhibition (Fig. 7a). Thus, miR-204 expression inhibits autophagy mediated degradation pathway in medulloblastoma cells.

### *TRPM3/MIR204* promoter methylation analysis and upregulation of miR-204 expression upon treatment with HDAC inhibitors

**Fig. 8:**
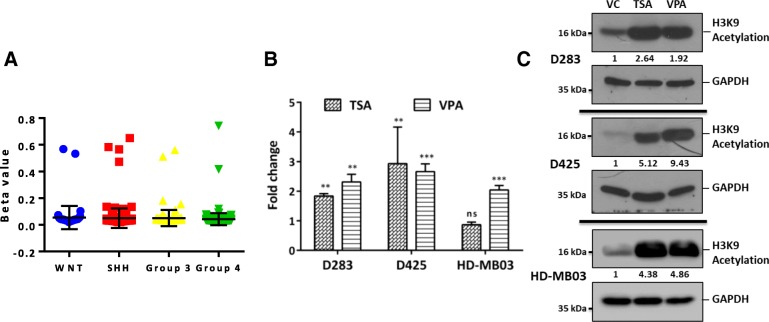
*TRPM3*/MIR204 promoter methylation analysis and upregulation of miR-204 expression upon treatment with HDAC inhibitors in medulloblastoma cells. **a**. Genome wide methylation data of the MAGIC cohort of 763 medulloblastomas was analyzed for methylation at the CpG island at the *TRPM3* / MIR204 promoter. The scatter plot shows the Beta values for probe cg23553442 located in the CpG island in the four molecular subgroups. **b**. Y axis denotes fold change in the expression levels of miR-204 after treatment of the indicated medulloblastoma cell line with HDAC inhibitors Sodium valproate (VPA) and Trichostatin A (TSA). **c**. Western blot analysis showing H3K9 acetylation levels in the indicated medulloblastoma cell line in the vehicle control (VC) and the HDAC inhibitor-treated cells. The numbers below the western blots indicate the fold change in the indicated protein level upon miR-204 expression using GAPDH as a house-keeping control. **, ***, ns indicate *p* ≤ 0.001, *p* ≤ .0001 and non-significant respectively

MiR-204 is located within the cancer associated genomic region at 9q21.1-q22.3 that exhibits high frequency of loss of heterozygosity in various cancers [55]. Loss of chromosome 9q is however, not frequent in Group 3 / Group 4 medulloblastomas with less than 20% and 5% loss of chr 9q arm in Group 3, Group 4 tumors respectively based on the analysis of structural variations in 1000 medulloblastomas [38]. Downregulation of miR-204 expression has also been reported to occur as a result of promoter methylation [58]. CpG island at the *TRPM3*/MIR204 promoter locus seems to be not methylated in Group 3 / Group 4 medulloblastomas based on the data from the Illumina 450 K array and bisulfite sequence analysis done on Group 3 medulloblastoma cell lines for the CpG island in the *TRPM3/*MIR204 promoter region (Fig. 8a; Additional file 4: Figure S3). HDAC inhibitors have been reported to inhibit growth of medulloblastoma cells particularly that of *MYC*-driven Group 3 cell lines [29, 43]. Treatment of medulloblastoma cell lines D283, D425 and HD-MB03 with Trichostatin A and Sodium valproate, the HDAC inhibitors resulted in 2 to 4 fold increase in expression levels of miR-204 (Fig. 8b) accompanied by the increased histone acetylation (Fig. 8c). Thus, treatment of medulloblastoma patients with HDAC inhibitors could help in upregulation of miR-204 that has tumor suppressive effect.

## Discussion

In a large scale study on 3312 tumors and 1107 non-malignant tissues contributed by 51 different cancer types, miR-204-211 family was found to be the top deleted microRNA family in cancer, suggesting its crucial role as a tumor suppressive miRNA in multiple cancer types [55]. In the present study, miR-204 was found to be differentially expressed in the four core molecular subgroups of medulloblastomas, with almost all SHH and a subset of Group 3/Group 4 tumors showing downregulation of miR-204 expression. Despite the complexity of the heterogeneity and overlap present in the copy number variations, methylation profiles and somatic mutation profiles in the Group 3 / Group 4 medulloblastomas [2], miR-204 expression levels identify a subset of these tumors having poor survival in the Indian as well as in the large MAGIC cohort. This finding is consistent with lower expression of miR-204 correlating with poor survival in breast cancer [23], non-small cell lung cancer [15], and neuroblastoma [47]. Integrated genomic studies have identified novel molecular subtypes within the four core subgroups of medulloblastomas [2, 49]. Among the three Group 3 subtypes, subtype 3γ was found to have the worst 5 year survival rate of 41.9% as compared to that of subtype 3α and subtype 3β at 66.2 and 55.8% respectively [2]. Group 3γ having the worst survival showed the least miR-204 expression among the 3 subtypes of Group 3. The Group 4 subtypes do not show significant difference in their overall survival [2]. MiR-204 expression on the other hand, identified a subset of Group 4 medulloblastomas having significantly poor survival of 59.7% as compared to 80% of the ‘miR-204 high’ subset. Group 3 poor prognostication markers like *MYC* amplification and isochrome 17q do not have prognostication value in Group 4 [44]. FSTL5 immunopositivity serves as a marker for poor prognostication in both Group 3 and Group 4 medulloblastomas [45]. Adult Group 4 patients have also been reported to have poor survival rates [46]. Loss of chromosome 11 or gain of chromosome 17 identify a small subset of Group 4 patients who have excellent survival [51]. Biology underlying these cytogenetic alterations is however, not understood. Thus, low miR-204 expression serves as a marker of poor prognosis in Group 4 that has paucity of markers for prognostication. Integrated genomic analysis is expensive as well as technically demanding and thus cannot be used in routine clinical practice for risk stratification. MiR-204, a single microRNA on the other hand, can be easily combined with the Nanostring assay that classifies medulloblastomas into the four molecular subgroups [39]. Furthermore, miR-204 due its small size resists degradation during formalin fixation and thus would be a reliable marker even in poor quality FFPE tissues.

Downregulation of miR-204 expression with poor survival is consistent with its tumor-suppressive effect in medulloblastoma cell lines. Restoration of miR-204 expression in multiple established Group 3 medulloblastoma cell lines was found to inhibit their anchorage-independent growth, invasion potential and tumorigenicity. Tumor suppressive effect of miR-204 in the *MYC* amplified Group 3 cell lines is remarkable since other microRNAs downregulated in medulloblastoma like miR-206 for instance, fail to inhibit tumorigenicity of these cell lines [41]. MiR-204 has been shown to inhibit invasion and tumorigenicity of various cancer cells including glioma, colorectal cancer, endometrial cancer and cervical cancer cells [3, 27, 57, 58]. Thus, the tumor suppressive role of miR-204 in medulloblastoma cells is consistent with its role in other cancers.

MiR-204 has been reported to target a number of genes including *RAB22A, FOXC1, EZR, BCL2L2, M6PR, BCL2, MCL1, FOXA1, FOXM1, EPHB2* [22, 42, 50, 52, 57]. Transcriptome sequencing / real time RT-PCR / western blot analysis showed downregulation of *RAB22A, M6PR, EZR, EPHB2,* upon miR-204 expression in medulloblastoma cells as well. *IGF2R* was identified and validated as a novel target of miR-204. MiR-204 expression in medulloblastoma cells resulted in downregulation of both M6PR and IGF2R that mediate transport of lysosomal enzymes from the Golgi apparatus to lysosomes [26]. Furthermore, reduction in the levels of lysosomal enzymes Cathepsin B and Cathepsin D upon miR-204 expression in medulloblastoma cells suggests impairment of the lysosomal degradation pathway. Autophagy brings about p62/SQSTM1 mediated degradation of its cargo by lysosomal degradation pathway [12]. MiR-204 is known to target LC3B, a crucial mediator of autophagy [28]. In the present study as well, miR-204 expression in medulloblastoma cells resulted in reduction in the LC3B flux and increase in the levels of p62/SQSTM1 indicating autophagy inhibition. Autophagy has been shown to play role in tumor promotion by sustaining survival in stress, by reducing oxidative stress and, maintaining metabolic homeostasis [14]. Inhibition of tumor growth upon miR-204 expression is consistent with these reports on the role of autophagy in tumor promotion. Autophagy has also been reported to promote invasion by activating Epithelial Mesenchymal Transition of hepatocellular carcinoma cells [48], by promoting secretion of factors like IL6, MMP2 [24] and by activating the MAP kinase signaling pathway in glioblastoma cells [8]. Consistent with the inhibition of invasion capacity of medulloblastoma cells upon miR-204 expression, downregulation of miR-204 expression was found to be associated with higher incidence of metastasis at diagnosis in Group 3 / Group 4 medulloblastomas. Thus, poor survival of Group 3 / Group 4 medulloblastomas having low miR-204 expression is likely due to their higher invasive capacity and higher malignant potential.

Several microRNAs whose expression is deregulated in medulloblastoma are known to play role in embryonic brain development [56]. MiR-9 and miR-124a that play crucial role in the onset of neurogenesis by targeting transcription factors like SOX9, FOXG1 and MEIS1, are downregulated in medulloblastoma [6]. MiR-9 and miR-199b-5p target HES1, thereby silence Notch signaling pathway at the onset of neuronal differentiation [7, 10]. Low expression of miR-9 and miR-199b-5p has been found to correlate with poor survival in medulloblastoma and their expression in medulloblastoma cell lines promotes growth arrest [7, 10]. MiR-17-92 cluster microRNAs are overexpressed predominantly in the SHH subgroup medulloblastomas [35]. Knock-out of this microRNA cluster brings about reduction in size of cerebellum and inhibits medulloblastoma formation in *Ptch* knock-out mouse model of SHH subgroup medulloblastomas indicating role of these microRNAs in normal development and tumorigenesis [33]. MiR-204 has been reported to play crucial role in lens and retinal development by targeting MEIS2 transcription factor in Medaka fish [4]. MiR-204 expression has been found to be upregulated during aging in mouse hippocampus and target Ephrin B2 that plays role in axon guidance [31]. MiR-204 has also been reported to control neuronal migration and cortical morphogenesis in mouse embryos presumably by targeting Doublecortin that is known to play role in neuronal migration [54]. Effect of miR-204 on invasive capacity of medulloblastoma cells is consistent with the role of miR-204 in neuronal migration. Thus, miR-204 appears to play role in both normal brain development and tumorigenesis like several other miRNAs that are known to be deregulated in medulloblastoma.

Delineating the molecular mechanism underlying downregulation of miR-204 expression would suggest ways to increase its expression, thereby improving survival rate of medulloblastoma patients. Group 3 medulloblastoma cells treated with HDAC inhibitors showed modest 2 to 4 fold increase in the miR-204 expression levels. Treatment with HDAC inhibitors has been reported to inhibit medulloblastoma cell growth in several studies [20, 29, 43]. Thus, HDAC inhibitors appear to have therapeutic potential in the treatment of medulloblastoma.

## Conclusions

In summary, downregulation of miR-204 expression correlates with poor survival in the Group 3 / Group 4 medulloblastomas. Furthermore, within the Group 4 itself, low expression of miR-204 identifies a subset having significantly poor survival, making it a valuable marker for risk stratification in the subgroup that has paucity of prognostication markers. Restoration of miR-204 expression leading to reduction in the invasive capacity and tumorigenic potential of medulloblastoma cells suggests therapeutic potential of miR-204 in the treatment of medulloblastomas. Upregulation of miR-204 expression upon treatment with HDAC inhibitors, although modest, suggests a role of these inhibitors in the treatment of medulloblastomas.

## Additional files

Additional file 1: **Table S1**. The nucleotide sequences of the primers used in the study. All sequences are given in 5′ to 3′ direction. (DOCX 15 kb)

Additional file 2: **Figure S1**. MiR-204 expression levels in the 8 subtypes of Group 3 / Group 4 medulloblastomas from Northcott et al. [34] data. (PPTX 39 kb)

Additional file 3: **Figure S2**. Kaplan Meier Survival Analysis of Group 3 medulloblastomas from the Indian cohort comparing overall survival of ‘miR-204 high’ subset with that of ‘miR-204 low’ subset. (PPTX 69 kb)

Additional file 4: **Figure S3**. Methylation analysis of the CpG island from the promoter region of *TRPM3*/MIR204. A 203 bp region of the CpG island from the promoter region of the *TRPM3* gene was PCR amplified from bisulfite converted genomic DNA of the medulloblastoma cell lines. Representative nucleotide sequence of this PCR product from the indicated medulloblastoma cell line is shown. Arrows indicate the CpG residues in the DNA sequence and their sequence in the bisulfite converted (BSP) DNA from the medulloblastoma cells. (PPTX 121 kb)

## Abbreviations

3′-UTR: 3′- untranslated region
ANOVA: Analysis of variance
ATCC: American Type Culture Collection
CI: Confidence interval
DMEM: Dulbecco’s Modified Eagle Medium
FBS: Fetal Bovine Serum
FFPE: Formalin- Fixed, Paraffin-Embedded
GSEA: Gene Set Enrichment Analysis
HDAC: Histone deacetylase
MAGIC: Medulloblastoma Advanced Genomics International Consortium
RMA: Robust Multi-array Average
RQ: Relative Quantity
RT-PCR: Reverse Transcription-Polymerase Chain Reaction
SDS-PAGE: Sodium Dodecyl Sulphate-Polyacrylamide Gel Electrophoresis
STR: Short Tandem Repeat
