# Supplementary material for: Downregulation of miR-204 expression defines a highly aggressive subset of Group 3/Group 4 medulloblastomas
Source: Acta Neuropathol Commun. 2019 Apr 3;7:52. doi: 10.1186/s40478-019-0697-3 (PMC6448261; doi:10.1186/s40478-019-0697-3)
Supplement: Supplementary file 1 — Table S1. The nucleotide sequences of the primers used in the study. All sequences are given in 5′ to 3′ direction. (DOCX 15 kb) [file 40478_2019_697_MOESM1_ESM.docx]

Table S1. The nucleotide sequences of the primers used in the study. All sequences are given in 5’ to 3’ direction.

| **Name of sequence** | **Forward** | **Reverse** |
| --- | --- | --- |
| **Primers used for amplification of genomic region encoding hsa-miR-204** | CTCCGGATCCTTTACCCACAGGACAGG | GAGGCTGCAGTAACCCCATCGTTAAGCA |
| **Primers used for Real Time PCR** | | |
| **RAB22A** | TGTAAGAGAAGTCATGGAGAGAGAT | CAGGTTGGCGTCAGTGGAT |
| **M6PR** | CAGTTTCCCACGACACGATG | GCCAGGAGTAGTAGTAGCA |
| **Primers used for cloning 3’UTR region** | | |
| **IGF2R 3’UTR** | GACGGATCCGCACCTCCAACCAAATAAGACT | GTCCTCGAGCCTATCGGGACTAAAGCAGC |
| **Primers used for site directed mutagenesis for mutating miR-204 binding site in 3’UTR region** | | |
| **IGF2R 3’UTR SDM** | CTTTAACAGAAACTTTCAAATATAAAGAGTTTTTGTGATGGGGGAG | CTCCCCCATCACAAAAACTCTTTATATTTGAAAGTTTCTGTTAAAG |

| **Primers used for bisulphite sequencing** | | |
| --- | --- | --- |
| **TRPM3/MiR-204 Promoter** | GGGTGGAGAGTAATTTGGGG | R1- CGAATCTCCCTCCAACCTA  R2- TTAATCTCCCTCCAACCTA |
