# Supplementary figures and images for: Downregulation of miR-204 expression defines a highly aggressive subset of Group 3/Group 4 medulloblastomas
Source: Acta Neuropathol Commun. 2019 Apr 3;7:52. doi: 10.1186/s40478-019-0697-3 (PMC6448261; doi:10.1186/s40478-019-0697-3)

## Slide 1
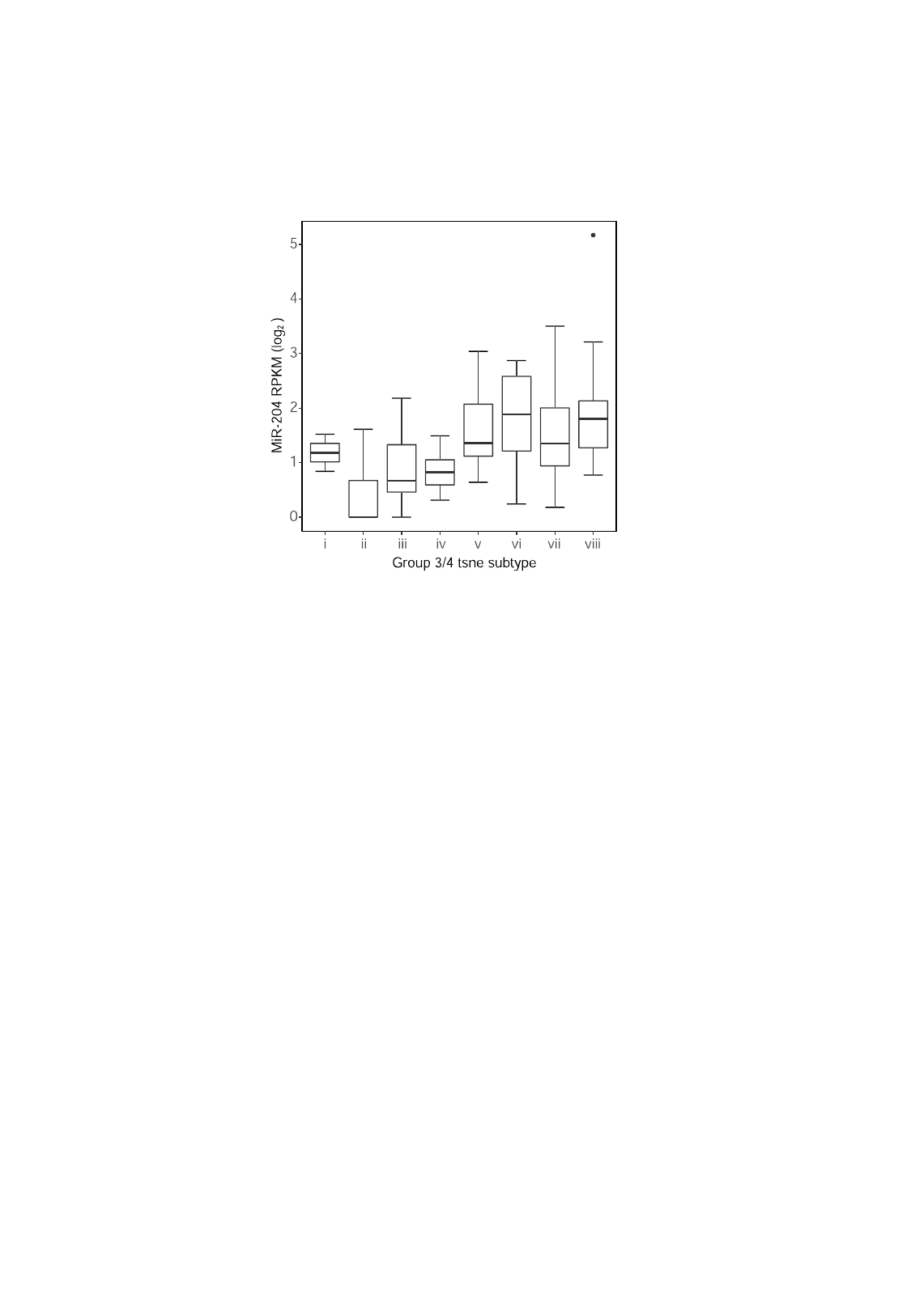

Supplement: Supplementary file 2 — Figure S1. MiR-204 expression levels in the 8 subtypes of Group 3 / Group 4 medulloblastomas from Northcott et al. [34] data. (PPTX 39 kb) [file 40478_2019_697_MOESM2_ESM.pptx]

## Slide 1
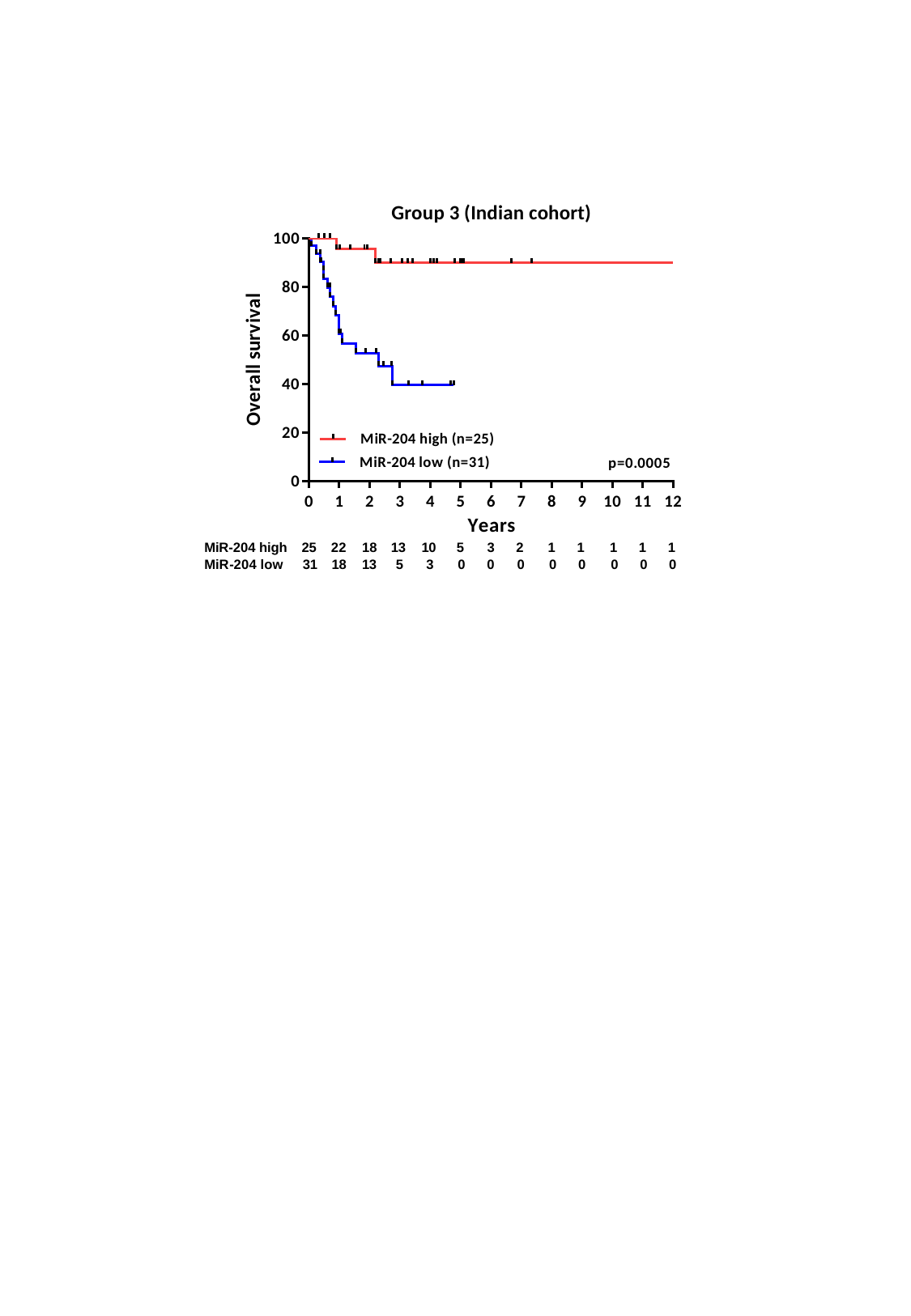

Supplement: Supplementary file 3 — Figure S2. Kaplan Meier Survival Analysis of Group 3 medulloblastomas from the Indian cohort comparing overall survival of ‘miR-204 high’ subset with that of ‘miR-204 low’ subset. (PPTX 69 kb) [file 40478_2019_697_MOESM3_ESM.pptx]

## Slide 1
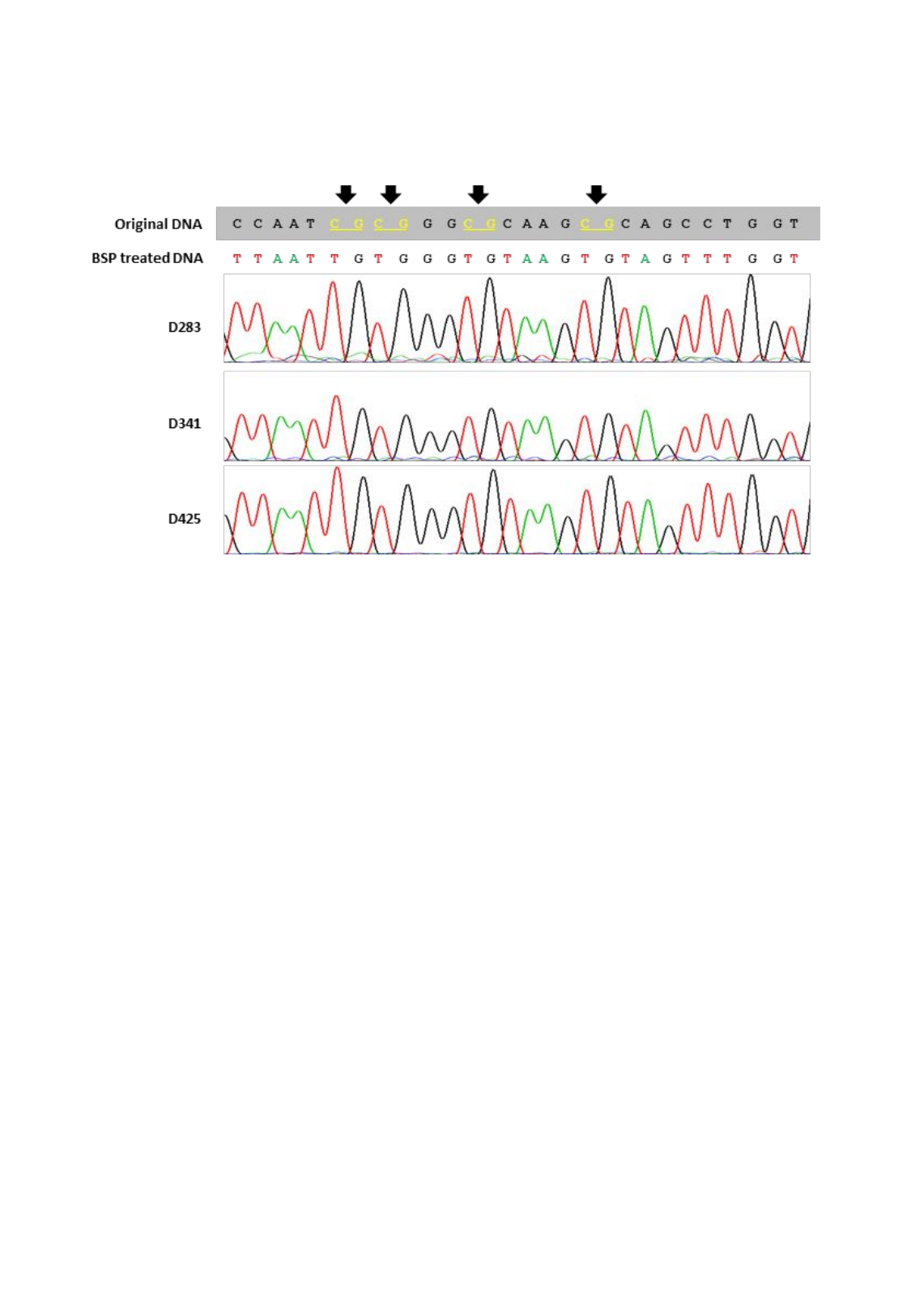

Supplement: Supplementary file 4 — Figure S3. Methylation analysis of the CpG island from the promoter region of TRPM3/MIR204. A 203 bp region of the CpG island from the promoter region of the TRPM3 gene was PCR amplified from bisulfite converted genomic DNA of the medulloblastoma cell lines. Representative nucleotide sequence of this PCR product from the indicated medulloblastoma cell line is shown. Arrows indicate the CpG residues in the DNA sequence and their sequence in the bisulfite converted (BSP) DNA from the medulloblastoma cells. (PPTX 121 kb) [file 40478_2019_697_MOESM4_ESM.pptx]
